# Open-Label Crossover Study of Primaquine and Dihydroartemisinin-Piperaquine Pharmacokinetics in Healthy Adult Thai Subjects

**DOI:** 10.1128/AAC.03704-14

**Published:** 2014-12

**Authors:** Borimas Hanboonkunupakarn, Elizabeth A. Ashley, Podjanee Jittamala, Joel Tarning, Sasithon Pukrittayakamee, Warunee Hanpithakpong, Palang Chotsiri, Thanaporn Wattanakul, Salwaluk Panapipat, Sue J. Lee, Nicholas P. J. Day, Nicholas J. White

**Affiliations:** a Faculty of Tropical Medicine, Mahidol University, Bangkok, Thailand; b Mahidol Oxford Tropical Medicine Research Unit, Faculty of Tropical Medicine, Mahidol University, Bangkok, Thailand; c Centre for Tropical Medicine, Nuffield Department of Medicine, University of Oxford, Oxford, United Kingdom

## Abstract

Dihydroartemisinin-piperaquine is an artemisinin-based combination treatment (ACT) recommended by the WHO for uncomplicated Plasmodium falciparum malaria, and it is being used increasingly for resistant vivax malaria where combination with primaquine is required for radical cure. The WHO recently reinforced its recommendations to add a single dose of primaquine to ACTs to reduce P. falciparum transmission in low-transmission settings. The pharmacokinetics of primaquine and dihydroartemisinin-piperaquine were evaluated in 16 healthy Thai adult volunteers in a randomized crossover study. Volunteers were randomized to two groups of three sequential hospital admissions to receive 30 mg (base) primaquine, 3 tablets of dihydroartemisinin-piperaquine (120/960 mg), and the drugs together at the same doses. Blood sampling was performed over 3 days following primaquine and 36 days following dihydroartemisinin-piperaquine dosing. Pharmacokinetic assessment was done with a noncompartmental approach. The drugs were well tolerated. There were no statistically significant differences in dihydroartemisinin and piperaquine pharmacokinetics with or without primaquine. Dihydroartemisinin-piperaquine coadministration significantly increased plasma primaquine levels; geometric mean ratios (90% confidence interval [CI]) of primaquine combined versus primaquine alone for maximum concentration (*C*_max_), area under the concentration-time curve from 0 h to the end of the study (AUC_0–last_), and area under the concentration-time curve from 0 h to infinity (AUC_0–∞_) were 148% (117 to 187%), 129% (103 to 163%), and 128% (102 to 161%), respectively. This interaction is similar to that described recently with chloroquine and may result in an enhanced radical curative effect. (This study has been registered at ClinicalTrials.gov under registration no. NCT01525511.)

## INTRODUCTION

The 8-aminoquinoline primaquine is the only available drug which rapidly and reliably kills mature gametocytes of Plasmodium falciparum and so limits transmissibility of the treated infection. The emergence of artemisinin resistance in P. falciparum infection and the drive to eliminate malaria in some areas where it is endemic have led the WHO to strengthen its recommendation to add primaquine as a gametocytocidal drug to all artemisinin-based combination treatments (ACTs) of falciparum malaria in these areas (1). A single primaquine dose of 0.75 mg base/kg of body weight or 45-mg adult dose was recommended originally as a gametocytocide and used in several countries for many years (2), but a lower dose of 0.25 mg base/kg (15-mg adult dose) has recently been recommended (1). This dose appears to be equally effective in blocking transmission, with a lower risk of hemolysis in glucose-6-phosphate dehydrogenase (G6PD)-deficient patients (3). It is considered that this single low dose can be given safely to patients with unknown G6PD status (1).

The WHO recommends dihydroartemisinin-piperaquine, a fixed-dose combination of dihydroartemisinin and piperaquine phosphate, as one of the first-line ACTs for the treatment of uncomplicated P. falciparum malaria. The normal adult dose is 3 tablets of dihydroartemisinin-piperaquine (40 to 320 mg/tablet) given once daily for 3 days (2). Dihydroartemisinin-piperaquine is also being used increasingly for the treatment of vivax malaria, particularly in areas where chloroquine resistance is prevalent (4–7). Primaquine (in 14-day regimens) is required for the radical cure of Plasmodium vivax and Plasmodium ovale infections because of its unique hypnozoitocidal activity. As a result, the use of dihydroartemisinin-piperaquine and primaquine together, in the treatments of both falciparum and vivax malaria, is likely to increase. Potential pharmacokinetic interactions have not been investigated. The pharmacokinetics of single-dose primaquine and dihydroartemisinin-piperaquine were studied in healthy Thai adult volunteers in a prospective, randomized, crossover study.

## MATERIALS AND METHODS

### Subjects.

Sixteen healthy Thai adults (11 female, 5 male) between 18 and 60 years of age were recruited. They were nonsmokers and were judged healthy based on clinical history, physical examination, and baseline screening results in hematology, biochemistry, urinalysis, and electrocardiogram (ECG), with a corrected QT (QTc) (Fridericia) interval of <450 ms. Exclusion criteria included a history of drug allergy, alcohol or substance abuse, concomitant medication intake, G6PD deficiency as detected by Beutler's dye test, or positive HIV, hepatitis B, or hepatitis C serology. Female subjects were of nonchildbearing potential or, if of childbearing potential, had a negative serum pregnancy test and agreed to use effective contraceptive methods during the study. The study protocol was approved by the ethics committee of the Faculty of Tropical Medicine, Mahidol University (reference number TMEC 12–004, approval number MUTM 2012-009-01) and by the Oxford University Tropical Research Ethics Committee (OXTREC 58–11). The trial was registered at Clinical Trials.gov under number NCT01525511. Each volunteer was provided with an explanation of the study and signed a written informed consent before study entry.

### Sample size.

The sample size was based on predicted areas under the plasma concentration-time curves (AUCs). Taking 80 to 125% as the no-relevant-effect limits for primaquine exposure (with or without dihydroartemisinin), and assuming a within-subject coefficient of variation for primaquine AUC of 21% (8), a sample size of 16 subjects (8 per sequence) provided a statistical power of 80%. The within-subject coefficients of variation associated with dihydroartemisinin (9) and piperaquine (10) AUCs were less than that associated with primaquine, so a sample size of 16 subjects also provided a satisfactory power for assessment of the effects of primaquine on dihydroartemisinin and piperaquine exposure. The sample size calculation was based on one-sided testing with an α value of 5% and assumed a true ratio of unity.

### Randomization and study design.

Volunteers were randomized into two groups. The study was open labeled, so patients and staff were aware of the study drug being administered. The volunteers were admitted to the pharmacokinetic unit at the Hospital for Tropical Diseases the evening before the study began. Subjects were given a light standard meal (∼200 kcal with 8 g fat) 30 min before each drug dose and were allowed to eat 4 h after administration of the study drug. Water and/or soft drinks without caffeine were permitted 2 h postdose. Study drugs were taken orally with a glass of water. Vital signs were checked every 4 h after dosing. Both groups received 2 tablets of primaquine phosphate (15 mg base/tablet; Government Pharmaceutical Organization, Thailand) in the first admission. In the second admission, one group (*n* = 8) was given a single dose of 3 tablets of dihydroartemisinin-piperaquine (Eurartesim; Sigma-Tau Industrie Farmaceutiche Riunite S.p.A.) only (40 mg dihydroartemisinin/320 mg piperaquine phosphate per tablet), and the other group (*n* = 8) received a single dose of 2 tablets of primaquine together with 3 tablets of dihydroartemisinin-piperaquine, and vice versa in the third admission. The washout periods between doses were >1 week after primaquine and >8 weeks after dihydroartemisinin-piperaquine–containing treatments. Electrocardiograms were recorded at 0, 1, 2, 4, 8, 12, and 24 h postdose in each admission. Methemoglobin was measured at each pharmacokinetic blood sampling time (see below) using a noninvasive monitoring machine (Masimo pulse oximeter; SpMet).

### Pharmacokinetic sampling.

For the pharmacokinetic assessment of all drugs, blood samples (2 ml) were collected into fluoride-oxalate tubes at 0 (predose), 0.25, 0.5, 1, 1.5, 2, 3, 4, 6, 8, 10, 12, and 24 h and on day 3 (48 to 54 h). An indwelling catheter was used for the multiple serial blood collections from 0 to 12 h postdose. Additional blood samples were taken for piperaquine measurements on days 4, 7, 11, 15, 22, and 36. After collection, blood samples were centrifuged for 7 min at 2,000 × *g* at 4°C, and plasma was stored at −70°C or lower. All samples were transferred to the Department of Clinical Pharmacology, Mahidol-Oxford Tropical Medicine Research Unit, Bangkok, Thailand, for plasma drug measurements. The laboratory participates in the WorldWide Antimalarial Resistance Network (WWARN) quality control and assurance proficiency testing program with satisfactory performance (http://www.wwarn.org/toolkit/qaqc).

### Drug analysis.

Dihydroartemisinin and piperaquine plasma concentrations were quantified using high-performance liquid chromatography linked with tandem mass spectrometry according to previously published methods (11, 12). The limit of quantification was 2.0 ng/ml for dihydroartemisinin and 1.50 ng/ml for piperaquine, respectively. Primaquine and carboxyprimaquine plasma concentrations were quantified using solid-phase extraction and high-performance liquid chromatography with mass spectrometry detection (reference 13 and; unpublished data). The limits of quantification were 1.14 ng/ml and 4.88 ng/ml for primaquine and carboxyprimaquine, respectively. Three replicates of quality control samples at low, middle, and high concentrations were analyzed within each batch of clinical samples to ensure precision and accuracy during drug measurements. The total precision (i.e., relative standard deviation [SD]) for all drug measurements was <9.0% during drug quantification.

### Pharmacokinetic analysis.

Individual subject concentration-time data were evaluated using a noncompartmental analysis approach as implemented in WinNonlin version 6.3 (Pharsight Corporation, USA). The terminal elimination rate constant (λ_Z_) was estimated by log-linear best-fit regression of the observed plasma concentrations in the terminal elimination phase, without data point removal. Visual inspection of all concentration-time profiles were performed to ensure an adequate fit to the observed data. Total exposure up to the last measured concentration (AUC_0–last_) was calculated using the linear trapezoidal method for ascending concentrations and the logarithmic trapezoidal method for descending concentrations. Exposure was extrapolated from the last observed concentration to infinity by *C*_last_/λ_Z_ for each subject to compute total drug exposure (AUC_0–∞_). The terminal elimination half-life (*t*_1/2_) was estimated by ln 2/λ_Z_. The maximum plasma drug concentration (*C*_max_) and time to maximum concentration (*T*_max_) were taken directly from the observed data. The total apparent volume of distribution (*V_z_*/*F*) and oral clearance (CL/*F*) were computed individually according to the equations *V*_z_/*F* = dose/(λ_Z_ × AUC) and CL/*F* = dose/AUC, respectively.

Pharmacokinetic parameter estimates were compared between a single dose of each study drug administered alone and in combination using the Wilcoxon signed-rank test in STATA v.11. An analysis of variance (ANOVA) was carried out on the log-transformed pharmacokinetic exposure parameters *C*_max_, AUC_0–last_, and AUC_0–∞_ to assess the bioequivalence of the drug administered alone versus that in combination. The effects of coadministration, the sequence of administrations, and subjects were examined in an adjusted model. The point estimate of the geometric mean ratio and the residual variability from the ANOVA were used to calculate the 90% confidence intervals (CIs) around the mean. The U.S. FDA criteria for assuming no interaction when the drugs are coadministered were met if the confidence intervals (90% CI) for the geometric mean ratios were retained within 80% to 125% (14).

### Safety analysis.

Safety was analyzed based on adverse events (AEs), physical examination, vital signs, clinical laboratory parameters, 12-lead electrocardiogram (ECG) findings, and methemoglobin levels. A ≥30-ms change from baseline in QTc interval (using Fridericia's correction) was specified prospectively as clinically significant, and any subject with this change at any time point was noted.

The safety and tolerability of primaquine and dihydroartemisinin-piperaquine were assessed by using the Wilcoxon matched-pair signed-rank test for continuous variables or McNemar's exact test for categorical variables when drugs were given alone or in combination. The frequencies (%) of adverse events and serious adverse events, with particular attention to those of potential clinical concern, were presented by treatment group and reported by visit so that any effect of the addition of primaquine and reexposure to piperaquine could be assessed. All liver function test (LFT) parameters were also compared within each visit by treatment (to assess the addition of primaquine) using the Mann-Whitney U test and within groups (to assess reexposure to dihydroartemisinin-piperaquine) using the Wilcoxon matched-pair signed-rank test. Subjects were analyzed as treated.

## RESULTS

### Subjects.

Sixteen healthy subjects were enrolled in the study and divided into group A (3 male and 5 female subjects) and group B (2 male and 6 female subjects). There were no clinically significant differences in baseline characteristics between the groups (Table 1). All of the volunteers completed the study protocol and were included in both the safety and the pharmacokinetic analyses.

**TABLE 1:** Baseline characteristics of study participants

Characteristic	Group A^*a*^	Group B^*a*^	*P* value^*b*^
Age (yr)	32.0 (24–45)	35.0 (26–52)	0.56
Weight (kg)	62.2 (54–71.4)	65.5 (54.3–68.9)	0.64
Height (cm)	162.5 (157–175)	164.5 (154–170)	0.96
No. (%) male	3 (37.5)	2 (25.0)	1.00^*c*^
Aspartate transaminase level (U/liter)	15.5 (11–21)	17.5 (14–19)	0.56
Alanine aminotransferase level (U/liter)	14.5 (7–25)	13.5 (10–30)	0.87
QTc interval (ms)^*d*^	415 (394–431)	413 (391–439)	1.00
Methemoglobin (%)	1.0 (0–1.6)	1.2 (0.6–1.3)	0.56

a Values are shown as median (range) unless noted otherwise. *n* = 8 per group.

b Mann-Whitney U test.

c Fisher's exact test.

d Fridericia's equation.

### Safety analysis.

The drugs were well tolerated. No clinically significant changes in the physical examination, vital signs, and clinical laboratory parameters were observed during the course of the study. QTc intervals (Fridericia) at the predose point of each regimen and their changes from before dosing at each time point up to 24 h are shown in Table 2. There was a small (median, 2%) but significant lengthening of the QTc (Fridericia) interval following dihydroartemisinin-piperaquine treatment with (8 ms) or without (7 ms) primaquine coadministration, which was maximal at 4 h after dosing compared to primaquine alone (*P* = 0.0009 and *P* = 0.0027, respectively). This correlated with the piperaquine *C*_max_ (correlation coefficient for maximum QTc prolongation following dihydroartemisinin-piperaquine alone [Kendall's tau] = 0.48, *P* = 0.01; in combination with primaquine, tau = 0.35, *P* = 0.0649). The addition of primaquine to dihydroartemisinin-piperaquine did not affect the magnitude of QTc prolongation (*P* = 0.5695) (Table 3). Two female subjects (38 and 31 years old) had a QTc interval marginally above 450 ms (450.3 and 450.51 ms, respectively) at 4 h after dihydroartemisinin-piperaquine administration. QTc interval prolongations from a predose baseline of >30 ms (32 and 33 ms, respectively) were observed in 2 subjects 4 h after dihydroartemisinin-piperaquine administration. All subjects had methemoglobin levels of <3% at all times during the study. Three severe adverse events (SAEs) were reported by 3 subjects. All were deemed unrelated to the study drug or study procedure. One subject had a rickettsial infection, 1 subject had unstable angina with dizziness with nonspecific ECG changes, and the third subject had acute bronchitis. All of the SAEs required hospitalization, and all resolved subsequently. Six other minor AEs were reported by 5 subjects and were considered unrelated to the study drug. All AEs resolved subsequently.

**TABLE 2:** QTc intervals (Fridericia's correction) at predose of each regimen and changes from before dosing to 24 h afterward

Dosing time	QTc change (ms) for treatment with^*a*^:			*P* value^*b*^ for:		
	Primaquine alone	DHA-PQP alone	Combination	Primaquine versus DHA-PQP	Primaquine versus combination	DHA-PQP versus combination
Predose	417.9 (17.9)	420.4 (13.7)	414.4 (14.8)	0.264	0.265	0.063
1 h^*c*^	−7.91 (−20.8 to −2.00)	−1.66 (−10.0 to 0.41)	−3.55 (−9.56 to 1.24)	0.088	0.017	0.959
2 h	−10.4 (−20.7 to −0.59)	−1.58 (−11.5 to 4.40)	0.06 (−7.22 to 5.54)	0.163	0.007	0.326
4 h	−3.37 (−7.98 to 4.97)	7.08 (2.16 to 22.1)	8.28 (2.76 to 14.6)	0.002	0.004	0.918
8 h	−14.2 (−19.9 to −7.84)	−0.001 (−10.8 to 3.36)	3.79 (−8.74 to 9.00)	0.023	0.020	0.408
12 h	−8.85 (−17.6 to −2.45)	−0.52 (−10.3 to 6.76)	−0.31 (−8.20 to 5.26)	0.034	0.070	0.717
24 h	−8.09 (−13.3 to −2.85)	2.99 (−9.93 to 9.95)	−0.95 (−2.42 to 12.0)	0.030	0.004	0.234

a *n* = 16 per treatment group. Values are shown as median (interquartile range) or mean (SD). DHA-PQP, dihydroartemisinin-piperaquine; combination, primaquine plus dihydroartemisinin-piperaquine.

b Compared using paired *t* test for predose and Wilcoxon matched-pair signed-rank test for all others.

c Missing data for 1 patient in primaquine group.

**TABLE 3:** The maximum electrocardiograph QTc (Fridericia's correction) readings within 24 h after drug administration, time of onset and changes from baseline

QTc reading	Treatment^*a*^			*P* value for:		
	Primaquine alone	DHA-PQP alone	Combination	Primaquine versus DHA-PQP	Primaquine versus Combination	DHA-PQP versus Combination
Time to onset (h)	4 (1–24)	4 (2–24)	4 (2–24)	0.0387	0.1474	0.7266
% change from predose	0.53 (−3.85 to 2.07)	2.10 (−1.85 to 8.13)	2.73 (−0.22 to 8.30)	0.0027	0.0009	0.5695

a Values are shown as median (range). *n* = 16 per treatment group. DHA-PQP, dihydroartemisinin-piperaquine; combination, primaquine plus dihydroartemisinin-piperaquine.

### Pharmacokinetic analysis.

**FIG 1:**
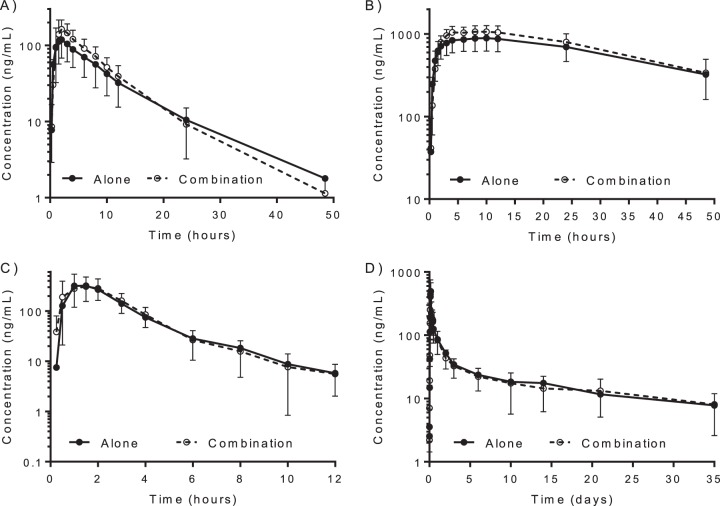
Mean venous plasma concentration-time curves of primaquine (A), carboxyprimaquine (B), dihydroartemisinin (C), and piperaquine (D) in healthy volunteers. Error bars indicate SDs.

**FIG 2:**
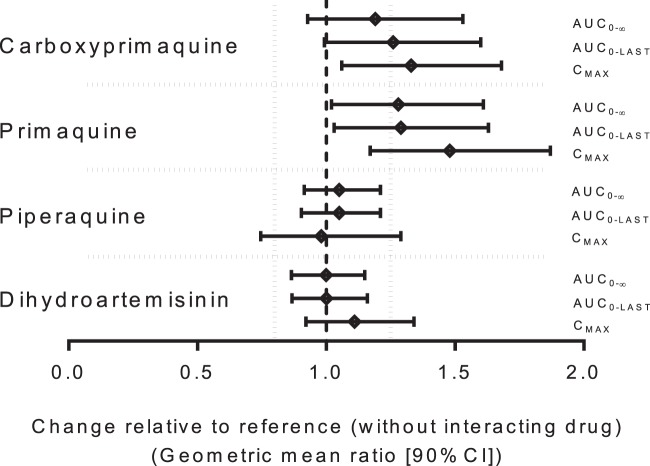
Forest plots of the geometric mean ratios (90% CI) of the drug administered with and without interacting drug for the logarithmically transformed *C*_max_, AUC_0–last_, and AUC_0–∞_. Vertical dashed lines represent the U.S. FDA criteria of 80 to 125% for assuming bioequivalence.

There were no statistically significant differences in dihydroartemisinin and piperaquine pharmacokinetics when administered with or without primaquine (Fig. 1; Table 4). The geometric mean ratios and 90% CIs of dihydroartemisinin and piperaquine administered with and without primaquine for the logarithmically transformed AUC_0–last_ and AUC_0–∞_ values were within the limits accepted for bioequivalence (Table 5; Fig. 2). However, the variability in *C*_max_ values was too great to assume bioequivalence.

**TABLE 4:** Pharmacokinetic parameters of dihydroartemisinin and piperaquine administered alone and in combination with primaquine

Parameter^*a*^	Dihydroartemisinin^*b*^			Piperaquine^*b*^		
	Alone	Combination	*P* value	Alone	Combination	*P* value
Total dose (mg/kg)	1.87 (1.68–2.22)	1.87 (1.68–2.22)	NA	8.65 (7.76–10.3)	8.65 (7.76–10.3)	NA
*C*_max_ (ng/ml)	364 (184–792)	348 (194–961)	0.3011	491 (129–1,270)	397 (127–1,200)	1.0000
*T*_max_ (h)	1.50 (1.00–2.00)	1.50 (0.50–3.00)	1.0000	4.00 (3.00–4.00)	4.00 (3.00–6.00)	0.7419
CL/*F* (liters/h/kg)	2.21 (0.96–5.01)	2.23 (0.87–5.52)	0.6051	0.450 (0.17–0.73)	0.441 (0.275–0.554)	0.1477
*V*/*F* (liters/kg)	5.53 (2.67–11.3)	5.89 (2.70–11.0)	0.1788	225 (120–593)	265 (139–339)	1.0000
*t*_1/2_ (h)	1.97 (1.13–2.67)	1.81 (1.13–2.84)	0.1788	390 (224–669)	449 (206–610)	0.6417
AUC_0–last_ (ng · h/ml)	812 (394–2,010)	890 (358–2,210)	1.0000	17,400 (8,120–36,800)	15,400 (12,200–31,200)	0.7960
AUC_0–∞_ (ng · h/ml)	817 (398–2,030)	899 (361–2,250)	0.9176	20,400 (11,400–57,300)	19,800 (15,400–35,900)	0.6417
Ext. AUC (%)	1.45 (0.253–4.19)	1.12 (0.338–2.81)	0.0703	17.9 (6.72–35.7)	20.8 (4.95–35.2)	0.6051

a *C*_max_, maximum observed plasma concentration after oral administration; *T*_max_, observed time to reach *C*_max;_ CL, elimination clearance; *V*, apparent volume of distribution; *t*_1/2_, terminal elimination half-life; AUC_0–last_, total exposure up to the last measured concentration; AUC_0–∞_, predicted area under the plasma concentration-time curve after the last dose from zero time to infinity; Ext. AUC, percentage of AUC_0–∞_ extrapolated from the last observation to infinity.

b Data are presented as median (range). *n* = 16 per treatment group. NA, not available.

**TABLE 5:** Bioequivalence analysis of dihydroartemisinin, piperaquine, primaquine and carboxyprimaquine after administration of dihydroartemisinin-piperaquine and primaquine alone and in combination^*b*^

Parameter^*a*^	Dihydroartemisinin	Piperaquine	Primaquine	Carboxyprimaquine
*C*_max_ (ng/ml)	111 (92.1–134)	98.1 (74.6–129)	148 (117–187)	133 (106–168)
AUC_0–last_ (ng · h/ml)	100 (86.7–116)	105 (90.3–121)	129 (103–163)	126 (99.3–160)
AUC_0–∞_ (ng · h/ml)	99.9 (86.5–115)	105 (91.4–121)	128 (102–161)	119 (92.8–153)

a *C*_max_, maximum observed plasma concentration; AUC_0–last_, total exposure up to the last measured concentration; AUC_0–∞_, predicted area under the plasma concentration time curve after the last dose from zero time to infinity.

b Data are presented as geometric mean ratios expressed as percentages (90% CI).

There were significant changes in the pharmacokinetics of primaquine and its major metabolite carboxyprimaquine when administered with dihydroartemisinin-piperaquine (Fig. 1; Table 6). Combined administration with dihydroartemisinin-piperaquine resulted in significantly lower primaquine CL/*F* (*P* = 0.0229) and *V*/*F* (*P* = 0.0013) values than administration alone, leading to significantly higher *C*_max_ (*P* = 0.0019) and AUC_0–last_ (*P* = 0.0200) values. This also resulted in a shorter primaquine *t*_1/2_ (*P* = 0.0005) than with administration alone. Geometric mean ratios (90% CI) of primaquine administered with and without dihydroartemisinin-piperaquine for *C*_max_, AUC_0–last_, and AUC_0–∞_ were 148% (117 to 187%), 129% (103 to 163%), and 128% (102 to 161%), respectively (Fig. 2).

**TABLE 6:** Pharmacokinetic parameters of primaquine and carboxyprimaquine after primaquine administered alone and in combination with dihydroartemisinin-piperaquine

Parameter^*a*^	Primaquine^*b*^			Carboxyprimaquine		
	Alone	Combination	*P* value	Alone	Combination	*P* value
Total dose (mg/kg)	0.468 (0.42–0.556)	0.468 (0.42–0.556)	NA	0.495 (0.444–0.588)	0.495 (0.444–0.588)	NA
*C*_max_ (ng/ml)	128 (20.2–249)	177 (68.1–303)	0.0019	959 (104–1,250)	1,150 (746–1,550)	0.0032
*T*_max_ (h)	1.79 (0.5–3.00)	2.00 (1.00–3.00)	0.4044	8.00 (4.00–12.0)	9.00 (3.00–12.0)	0.4355
CL/*F* (liters/h/kg)	0.403 (0.173–2.98)	0.344 (0.190–0.742)	0.0200	0.0122 (0.00507–0.141)	0.0118 (0.00572–0.0196)	0.1089
*V*/*F* (liters/kg)	4.19 (2.46–19.3)	2.87 (2.02–4.86)	0.0013	0.418 (0.346–3.304)	0.341 (0.269–0.475)	0.0019
*t*_1/2_ (h)	6.78 (4.45–9.84)	5.55 (3.81–7.56)	0.0005	23.1 (15.6–56.9)	22.6 (14.6–38.4)	0.0084
AUC_0–last_ (ng · h/ml)	1,090 (144–2,750)	1,370 (592–2,550)	0.0200	30,800 (2,870–44,200)	33,600 (20,800–55,900)	0.0262
AUC_0–∞_ (ng · h/ml)	1,130 (149–2,830)	1,400 (604–2,570)	0.0299	44,000 (3,320–92,100)	46,500 (24,200–90,600)	0.2146
Ext. AUC (%)	1.95 (0.502–7.00)	1.01 (0.233–6.56)	0.0299	24.6 (13.2–56.8)	24.1 (11.2–43.1)	0.0151

a *C*_max_, maximum observed plasma concentration after oral administration; *T*_max_, observed time to reach *C*_max;_ CL, elimination clearance; *V*, apparent volume of distribution; *t*_1/2_, terminal elimination half-life; AUC_0–last_, total exposure up to the last measured concentration; AUC_0–∞_, predicted area under the plasma concentration time curve after the last dose from zero time to infinity; Ext. AUC, percentage of AUC_0–∞_ extrapolated from the last observation to infinity.

b Data are presented as median (range). *n* = 16 per treatment group. NA, not available.

Similarly, when primaquine was administered in combination with dihydroartemisinin-piperaquine, there were also significantly higher carboxyprimaquine exposures (*C*_max_, *P* = 0.0032; AUC_0- last_, *P* = 0.0262) and lower *V*/*F* (*P* = 0.0019) and shorter *t*_1/2_ (*P* = 0.0084) values than with administration alone. The geometric mean ratios (90% CI) of carboxyprimaquine administered with and without dihydroartemisinin-piperaquine for *C*_max_, AUC_0- last_, and AUC_0–∞_ were 133% (106 to 168%), 126% (99.3 to 160%), and 119% (92.8 to 153%), respectively (Fig. 2). This follows the pattern of alteration in primaquine pharmacokinetics, confirming a significant drug-drug interaction between primaquine and dihydroartemisinin-piperaquine.

## DISCUSSION

The values of the pharmacokinetic parameters estimated for dihydroartemisinin and piperaquine in this study are mostly comparable to those of a previous study by Chinh and coworkers (10) (geometric means of dihydroartemisinin: *T*_max_, 1.5 h; *t*_1/2_, 1.01 h; CL/*F*, 5.45 liters/h/kg; *V*/*F*, 7.97 liters/kg; geometric means of piperaquine: *T*_max_, 3.0 h; *t*_1/2_, 589 h; CL/*F*, 0.47 liters/h/kg; *V*/*F*, 394 liters/kg). The present study showed lower clearance and volume of distribution of dihydroartemisinin and consequently higher AUC and *C*_max_ values (geometric means: AUC_0–∞_, 817 versus 370 ng · h/ml; *C*_max_, 364 versus 159 ng/ml). The present study also showed a higher *C*_max_ but a similar AUC of piperaquine (*C*_max_, 491 versus 204 ng/ml; AUC_0–∞_, 20,400 versus 19,929 ng · h/ml). These differences observed between studies may reflect differences in the volunteers' age, diet, or gender and/or the play of chance given the large interindividual variability and small sample sizes. Piperaquine absorption may be enhanced when administered with a high-fat meal (15, 16), although small amounts of fat have little effect on piperaquine bioavailability (17, 18). In this study, no drug-drug interactions were observed in dihydroartemisinin and piperaquine pharmacokinetics as a result of primaquine coadministration. The AUCs of dihydroartemisinin and piperaquine were all within the 90% CI of the geometric means ratio of 80 to 125%.

When administered alone, primaquine pharmacokinetic results were comparable to those of the previous studies. Elmes et al. (19) reported mean (SD) plasma primaquine values in healthy Australian men and women, respectively, as follows: *C*_max_ of 93 (26) and 115 (38) ng/ml, AUC_0–∞_ of 1,105 (475) and 1,240 (444) ng · h/ml, and CL/*F* of 0.34 (0.12) and 0.39 (0.14) liters/h/kg. Binh et al. (20) reported a median plasma primaquine *C*_max_ of 122 ng/ml, *T*_max_ of 2.0 h, and *t*_1/2_ of 6.1 h in healthy Vietnamese volunteers. Coadministration of primaquine with dihydroartemisinin-piperaquine resulted in significantly higher exposure, higher *C*_max_, lower *V*/*F*, and shorter *t*_1/2_ of both primaquine and carboxyprimaquine.

This pharmacokinetic interaction is similar in direction to that recently observed with other antimalarials. Coadministration of primaquine with chloroquine and with pyronaridine-artesunate demonstrated similar changes in primaquine pharmacokinetics (13; unpublished observations). In studies conducted >60 years ago, mepacrine (quinacrine [Atabrine]), an acridine with structural similarities to chloroquine, markedly elevated levels of pamaquine (plasmoquine, an 8-aminoquinoline predecessor of primaquine) (21, 22). Thus, several structurally related antimalarials, all with extensive tissue distribution and very slow elimination, elevate plasma concentrations of the 8-aminoquinoline drugs. Tissue displacement is therefore one potential mechanism to explain the interaction, and the likely interacting drug is therefore piperaquine rather than dihydroartemisinin. Whether this involves competition for transporters, such as that demonstrated in a study on the effect of rifampin, an organic anion-transporting polypeptide (OATP) inhibitor, on digoxin metabolism in rats (23), remains to be determined. Inhibition of uptake to the liver could explain the discrepancy between CL/*F* and *V*/*F*. This scenario is based on the assumption that primaquine is passively absorbed from the gut but actively transported into hepatocytes. An increase in the bioavailability of primaquine seems less likely given that volunteer studies suggest near-100% oral bioavailability for primaquine (24). Primaquine metabolism involves monoamine oxidase A (25) and cytochrome P450 (CYP) isozymes, especially 2C19 (25), 2D6, and 3A4 (25, 26). Piperaquine inhibits CYP3A4 (27, 28) and CYP2C19 (27), and metabolic inhibition cannot be excluded as a contributor to reduced primaquine clearance. As the active metabolites of primaquine are produced via CYP2D6, a different route to the monoamine oxidase pathway which produces carboxyprimaquine, the relevance of these findings to primaquine's pharmacodynamic effects remains to be determined. However, by inference, the efficacy synergy for radical curative activity in P. vivax malaria for chloroquine and primaquine and the toxicity synergy demonstrated for pamaquine and mepacrine point to an increased pharmacodynamic effect as a result of these drug-drug interactions. Dihydroartemisinin is metabolized rapidly by glucuronidation (29) and is eliminated more rapidly than primaquine, so it is unlikely to have contributed to reduced primaquine clearance. Malaria infection (8) and the standard three-dose regimen of dihydroartemisinin-piperaquine may further affect this interaction (30).

The overall tolerability for the dihydroartemisinin-piperaquine and primaquine combination was good compared to that of dihydroartemisinin-piperaquine or primaquine alone. While a light meal before each drug administration may help reduce the gastrointestinal side effects of primaquine (31), this fed state is also suggested to increase dihydroartemisinin-piperaquine absorption (15, 16). It has been suggested that dihydroartemisinin-piperaquine should be taken on an empty stomach because of concerns over electrocardiographic QTc interval prolongation (27). Our study showed a slight QTc interval prolongation in some female subjects receiving dihydroartemisinin-piperaquine which correlated with piperaquine levels and was similar in magnitude to that shown in earlier studies (32, 33) and that associated with chloroquine (600-mg adult dose) (13). Primaquine did not affect the QTc interval prolongation in this or other studies. Of all nine AEs reported, none were considered drug related.

In conclusion, coadministration of dihydroartemisinin-piperaquine and primaquine was well tolerated in healthy adult subjects. This combination did not result in any significant pharmacokinetic alterations of dihydroartemisinin and piperaquine but increased plasma concentrations of primaquine. Further study is required to determine how this affects primaquine pharmacodynamics, but there seems to be no reason to not recommend this combination.
